# Revisiting the concept of Innovative Developing Countries (IDCs) for its relevance to health innovation and neglected tropical diseases and for the prevention and control of epidemics

**DOI:** 10.1371/journal.pntd.0006469

**Published:** 2018-07-12

**Authors:** Alexandre Guimarães Vasconcellos, Bruna de Paula Fonseca e Fonseca, Carlos Medicis Morel

**Affiliations:** 1 Postgraduate and Research Division, National Institute of Industrial Property (INPI), Rio de Janeiro, Rio de Janeiro, Brazil; 2 National Institute of Science and Technology for Innovation on Diseases of Neglected Populations (INCT-IDPN), Center for Technological Development in Health (CDTS), Oswaldo Cruz Foundation (Fiocruz), Rio de Janeiro, Rio de Janeiro, Brazil; Institute for Disease Modeling, UNITED STATES

## Abstract

**Introduction:**

Countries have traditionally been split into two major groups: developed or industrialized (“the North”) and developing or underdeveloped (“the South”). Several authors and organizations have challenged this classification to recognize countries that have reached an intermediate stage of social and economic development. As proposed by Morel and collaborators in 2005, the concept of Innovative Developing Countries (IDCs) defines a group of nations with impactful scientific programs. Here, IDCs are reexamined by a variety of metrics to highlight their role in health innovation through research and development (R&D) programs on neglected tropical diseases (NTDs) that also positively impact epidemic preparedness.

**Results:**

To address the global changes due to expanding globalization we updated the original indicator of the number of USPTO patents deposited by individual countries per GDP and per capita to the number of international patents applications, related to applicant residence and deposited under the Patent Cooperation Treaty (PCT) per GNI (or GDP) and per capita. A comparison of the originally described ranking of top innovative countries to those in the present study revealed new members that updated the list of IDCs and showed a prominent role now played by China.

Analyzing scientific publications in international journals since the introduction of the IDC concept in 2005 we found that IDCs do prioritize Neglected Tropical Diseases (NTDs) as an area of research.

Finally we investigated the role of IDCs in two major public health emergencies between 2012 and 2016, the outbreaks of Ebola in West Africa and Zika in South America. An analysis of the co-authorship country networks demonstrated an important role for IDC infrastructure and personnel in the prevention and control of these epidemics.

**Discussion and conclusions:**

Different techniques can be used to evaluate and measure innovative performance of countries. Country rankings published by traditional indexes, such as the Bloomberg Innovation Index (BII) and the Global Innovation Index (GII), only include high income economies among the top 20 performers. This is in sharp contrast to our approach, which identified 8-9 IDCs among the first 25 with China occupying the top position. Through an analysis of the pros and cons of the different methodologies, the IDC concept challenges more conventional approaches to address and estimate the innovative capacity of countries.

## Author summary

Splitting countries into two groups—rich and poor; developed (the “North”) and developing (the “South”); leaders and followers—appears to us to be progressively more simplistic, unrealistic and a heritage from colonial times. Triggered by the first wave of globalization, the share of world income going to today’s wealthy nations soared from twenty to almost seventy percent between 1820 and 1990, a fact that supported and strengthened this dichotomic vision; however, the new globalization driven by information technology has propelled the rapid industrialization of several developing nations and simultaneous deindustrialization of developed nations, a phenomenon that has not yet been fully understood nor reflected in traditional economic indexes and analyses. In this article we revisit the 2005 concept of Innovative Developing Countries (IDCs) that points to the underrepresentation of IDCs in well-known innovation indexes and country ranks. Our analysis clearly shows a prominent role for IDCs in health innovation, research and development on NTDs and in epidemics preparedness, prevention and control.

## Introduction

Countries have traditionally been divided into two broad categories according to their capacity to innovate: *leaders*, which have infrastructure along with the human and financial resources for the production and management of innovation; and *followers*, those that do not have the capacity for innovation and face the challenge to reproduce and absorb technologies from the leading countries through technology transfer processes.

**Fig 1:**
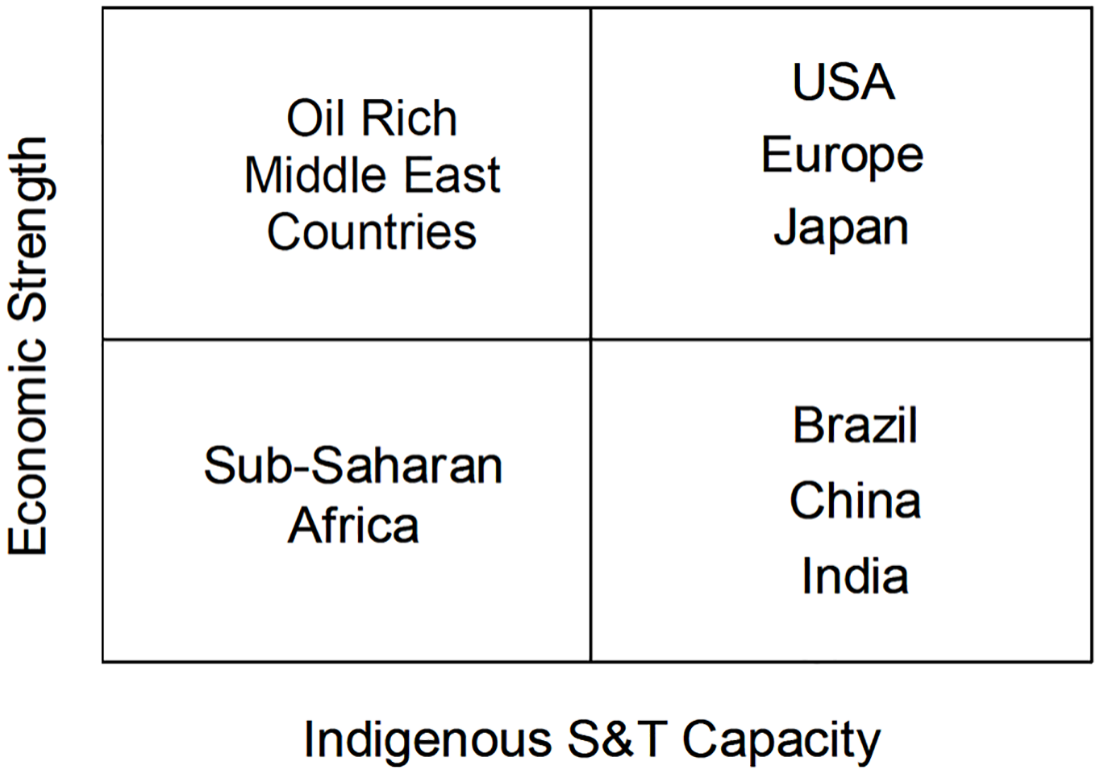
Distribution of countries according to R. A. Mashelkar. Developed, industrialized nations occupy the top-right quadrant; less developed countries the bottom-left quadrant. Countries of high economic strength due to abundant natural resources (such as the oil-exporters rich countries of the Middle East) occupy the top-left position. The lower-right quadrant was regarded by Mashelkar as the most interesting as it was home of countries with high S&T capacity but at the turn of the century relatively weak from an economic point of view. Reproduced from [1] with permission.

From analyzing this issue the Director General of the Council for Scientific and Industrial Research of India, R. A. Mashelkar (http://www.mashelkar.com/), proposed in 2003 an organization of countries in a 2x2 table that distributes them according to relative economic strength and autochthonous (or “indigenous” as he called it) science and technology (S&T) capacity Fig 1 [1]:

Mashelkar pointed out that the positions of the countries in these four quadrants should not be seen as static, citing the example of South Korea which in 1996 left the lower-right quadrant to become an OECD country.

To place the framework presented by Mashelkar into a quantitative basis, Morel and collaborators used the number of patents granted in the United States as a measure of the innovative capacity of a country when at least one inventor was from that country, which was correlated to the economic strength of the country based on economic and demographic criteria [2]. They designed a new indicator (number of patents normalized per GDP and per capita for each country) that made it possible to expand from Mashelkar’s vision of quadrants into rankings. Through this analysis, several countries that were allocated to the lower-right quadrant, which were not considered to have high income economies according to World Bank definitions, appeared among the top performers. The findings became the basis for the development of the concept of *Innovative Developing Countries* (IDCs) by Morel and collaborators that included Mashelkar [2, 3]. After a brainstorming meeting at the Bellagio Center, Rockefeller Foundation, 10-13 May 2004, it was agreed that:

“IDCs have the capacity to develop, manufacture, ensure safety, and market new health products and to develop, test and introduce new health polices or strategies. They are distinguished by their rapid growing strength in health innovation as illustrated by increasing patenting and publishing activities; increasing investments in technology by both the public and private sectors; rapidly growing number of health technology companies; and health systems able to analyze, evaluate and adopt new practices and technologies.”

“All developing countries can undertake health innovation to varying degrees. Some developing countries, however, are more scientifically advanced than others and are starting to reap benefits from decades of investments in education, health research infrastructure, and manufacturing capacity. We refer to these as innovative developing countries (IDCs)”

This perception that the term “developing countries” could not account for the diversity of countries based on innovation capacity was pioneering and visionary. For example, the 2016 edition of the World Development Indicators (WDI) published by the World Bank [4], no longer uses the terms “developed” and “developing” countries as analytical categories since recent evidence shows that dividing countries into just two groups does not reflect reality [5, 6]; instead, it adopts four categories (high income, upper-middle, lower-middle and low income economies). Vollmer and collaborators have recognized that today there is an emergence of “three human development clubs”, which is in contrast to the conceptual viewpoint of the 60s, when the world was clearly divided into industrialized and developing nations [7]. In the past, it was straightforward to split the countries into two groups when using indicators such as child mortality and fertility rate; today these indicators spread them along a continuum. Therefore, allocating a country into a single category does not necessarily contribute to understanding the present global realities.

In the area of health, it is particularly important to investigate how the research, development and innovation infrastructure built over the years by IDCs has been used to address neglected tropical diseases; furthermore whether this base could be mobilized for coping with new health challenges, such as specific epidemic situations.

This paper aims to revisit the concept of IDCs, thirteen years after its proposed use to define a new category of countries, and its relevance to health innovaton. Here, we address three topics: (i) review and update of the original country innovation ranking; (ii) relevance of IDCs in health research, development and innovation, particularly in relation to NTDs; (iii) role of IDCs that had invested in NTDs during the Ebola and Zika epidemics control and preparedness.

## Methods

To address the topics stated above we have structured the methodology along three main axes, describing for each one the approach adopted, the methods used and the calculations performed, as appropriate.

### Patent search and calculations: Update of the original country ranking

The innovation index proposed by Morel et al [2, 3] was originally obtained dividing the total number of patents filed in the United States by each country by their respective GDP per capita (S1 Table, Supporting Information). In the present study we refined this approach by dividing the total number of patents filed by each country under the Patent Cooperation Treaty (PCT) by their respective GNI or GDP per capita. Countries that were not considered high income economies by the World Bank (GNI per capita higher than US$ 12,476 in 2015) but in our analysis ranked among the top 25 innovative nations, fit the IDC category.

The search for patents filed in 2015 under the Patent Cooperation Treaty (PCT) was performed in July 2017 using Patentscope from the World International Property Organization (WIPO). Using the “field combination” tool, the query included the following fields: “WIPO publication number” (WO*), “Applicant residence” (XX*, where XX denoted country codes) and “Publication date” (01.01.2015 to 31.12.2015). To retrieve patents related to medicines, the additional field “International Class” was used to specify the patent subclass A61K* which according to International Patent Classification (IPC) refers to preparations for medical, dental or toilet purposes [8].

Population, Gross National Income (GNI) per capita and Gross Domestic Product (GDP) per capita of 2015 were obtained from the World Development Indicators DataBank [9].

### Bibliometric analysis: Relevance of IDCs in health research, development and innovation in NTDs

Scientific publications addressing at least one of the 17 NTDs listed by the World Health Organization were used as a representation of country focus on IDCs common health burdens.

Publications on NTDs were retrieved as raw data files from the Web of Science Core Collection (WoS) database. The total number of articles and articles on NTDs published by a given country during the 2005-2017 period were retrieved using the following profiles in “Advanced Search Mode”: (i) Total articles: cu = “name of country”; (ii) Articles mentioning at least one of of the NTDs in the abstract: cu = “name of country” AND ts = (“buruli ulcer” OR “Chagas disease” OR “trypanosoma cruzi” OR dengue OR chikungunya OR dracunculiasis OR echinococcosis OR “food borne trematobiases” OR “human african trypanosomiasis” OR “sleeping sickness” OR leishman* OR leprosy OR filariasis OR onchocerciasis OR “river blindness” OR rabies OR schistosomiasis OR helminthiasis OR taeniasis OR cysticercosis OR trachoma OR yaws OR “endemic treponomatoses”).

Countries were ranked according the decreasing percentage of articles mentioning at least one NTD in relation to the total number of articles published during the considered period.

### Co-authorship network analysis: Role of IDCs in the Ebola and Zika epidemics

Social network analysis (SNA) of scientific collaborations was pioneered by Newman [10, 11]. We used his approach as previously described [12–14] to investigate the role of IDCs in recent global epidemics.

Network analysis is a theoretical approach that employs a set of techniques used to understand and quantify the relationship between members of a network (nodes), which can be individuals, institutions, countries etc. [15]. By analyzing and quantifying the social structure of a network that is embedded in its nodes and connections, it is possible to assess different perspectives on the importance of individual nodes. In this work, we used the ‘betweeness centrality’ indicator to identify key countries that are frequently on the shortest paths between other countries, acting as intermediaries of information [16].

Articles published during the peak of the Ebola (n = 1,461 articles in 2015) and Zika (n = 1,477 articles in 2016) epidemics were retrieved from the Web of Science Core Collection (WoS) database as described above. The search query was directed to the title of the papers using the terms “Zika” or “Ebola”, accordingly. The unit of analysis (the nodes in the network) consisted of the countries where the authors were based at the time of publication, according to their affiliation data registered in each article. Only published or in press articles were included in the analysis. Publication data was imported into the data/text mining software VantagePoint (Search Technology Inc.).

After processing, data was formatted into adjacency matrixes [15] by VantagePoint to map co-authorship relationships between countries. Matrixes were imported into the open-source software Gephi [17] for network visualization and calculation of centrality metrics. For the spatial visualization of the international collaboration networks, country affiliation data were manually geocoded and processed using the “GeoLayout” and “Map of Countries” plugins available within Gephi. In these networks, nodes represent countries, and two or more countries were connected if their members shared the authorship of one or more papers. As co-authorship requires reciprocal cooperation among the participants, all connections have been considered as non-directional.

## Results

### IDCs, 2005-2017

In our present calculations we substituted the number of patent applications deposited at the USPTO, used in the original study [2], for the number of international patent applications deposited under the Patent Cooperation Treaty (PCT), related to applicant residence. This change addressed the global changes due to the continual progression of globalization [18]. In addition, the survey of the international markets instead of just the US market provided a broader view of the intent of patent protection of technologies.

We also explored the characteristics of our metrics in two additional ways. Firstly, using both the Gross National Income (GNI) per capita and the Gross Domestic Product (GDP) per capita. GDP measures total output produced, based on location, focused on domestic production and represents the strength of a country’s economy, while GNI measures the total income received, based on ownership, focused on income generated by citizens and represents economic strength of country’s nationals. We found that interchanging GDP for GNI did not significantly alter the final results and rankings (Supporting Information S2 Table). As we are particularly interested in the country residence of the patent applicant and in the income generated by the residents of that country, we adopted GNI per capita as the default. Secondly, using specific patent classes in the calculations, therefore selecting and delineating areas of technology or products of interest, allowed the detection of those countries that are more active in a given “product space” [19].

Table 1 displays the comparison of the 25 top innovative countries ranked as described in the original 2005 paper (first country column) and in this study (third country column). The second country column is an update of the first one using USPTO 2015 patent information. To demonstrate the potential and flexibility of our approach, the last column displays the country ranking and the leadership of India when only patents related to medicines are used in the calculations (patent subclass A61K see Methods) [20]).

**Table 1:** Top 25 innovative countries. Comparison of the 2005 original country ranking with those of the present study.

#	Morel et al, 2005 [2]	Ditto, updated	This study	Ditto, specific patents
	USPTO 2003 per GDP per capita	USPTO 2015 per GDP per capita	PCT 2015 per GNI per capita	PCT A61K 2015 per GNI per capita
1	USA	*India*	*China*	*India*
2	Japan	USA	Japan	*China*
3	*India*	*China*	USA	USA
4	*China*	Japan	*India*	Japan
5	Germany	Republic of Korea	Republic of Korea	Republic of Korea
6	Republic of Korea	Germany	Germany	France
7	France	Canada	France	Germany
8	Canada	UK	UK	United Kingdom
9	UK	France	Netherlands	*Russian Federation*
10	Italy	Israel	Italy	Switzerland
11	Israel	Italy	*Russian Federation*	Spain
12	*Brazil*	Netherlands	*Turkey*	*Brazil*
13	Sweden	*Russian Federation*	Sweden	Italy
14	*South Africa*	*Brazil*	Canada	Netherlands
15	Australia	Ukraine	*Brazil*	Canada
16	Switzerland	*Mexico*	Spain	*Turkey*
17	Belgium	Sweden	*South Africa*	Israel
18	Finland	Spain	Switzerland	Ukraine
19	Austria	Belgium	Ukraine	Belgium
20	*Thailand*	Switzerland	Israel	*Mexico*
21	*Argentina*	Australia	Finland	Poland
22	Singapore	*Philippines*	*Mexico*	*South Africa*
23	*Malaysia*	Austria	Austria	*Malaysia*
24	*Mexico*	*Malaysia*	*Malaysia*	Australia
25	*Indonesia*	Finland	Australia	*Kenya*

Countries that are not considered high income economies by the World Bank (GNI per capita higher than US$ 12,476) and fit the IDC category are displayed in italics. An example of the calculations using real data and also demonstrating that interchanging GDP for GNI does not alter significantly the final results can be found in Supporting Information S1 and S2 Tables.

Comparing the 2005 rank (first country column) with the results of the present study (third country column), some points are worth mentioning:

- The United States lost the first position, replaced by China;
- Japan retained the second position;
- The Russian Federation and Turkey are now in the list;
- Thailand, Argentina and Indonesia were removed from the list;
- Brazil and South Africa each lost 3 positions;
- India leads the ranking when (i) the original 2005 methodology is updated to USPTO 2015 or (ii) only the subset of patents related to medicines is considered.

### IDCs and neglected tropical diseases

In their original article Morel and collaborators addressed the innovative capacity of IDCs in health by analyzing patents that included the words “drug”, “vaccine”, or “pharmaceutical” in the abstract. It was observed that the rate of patenting was relatively constant during the first half of the 1990s, but accelerated dramatically after 1996 [2]. One important question however was not addressed at that stage: is the innovative capacity of IDCs being used to address health issues that are particularly relevant for their own populations or is it ‘market-driven’, and aimed at competing in the global markets? Or, as one participant said at the Bellagio meeting mentioned above: “Are the IDCs investing in their own health priorities or trying to develop the next blockbuster drug?”

Mahoney, Morel and collaborators [2, 21–24] identified six determinants, or components, of health technology innovation: (i) Development and expansion of national health delivery systems, including an attractive, domestic, private-sector market for health products; (ii) Development of manufacturing capability for health products; (iii) The drug and vaccine regulatory system; (iv) The IP regulatory system; (v) Development of R&D capability by the public and private sectors; (vi) Development of international trade systems for health products, including global procurement funds. Because these innovation components are dynamically linked, successfully developing and introducing new technologies requires concerted attention to each of the six components [22, 23]. Using this framework, Morel et al analyzed the progress of developing countries in innovation capability and identified three intermediate stages before reaching a similar development level of industrialized nations [2].

NTDs represent an enormous health burden for developing countries which only recently have been recognized as a global challenge meriting global efforts and commitments from public and private sectors [25, 26]. In order to investigate whether NTDs represents a priority for the research and technological development agenda of the top innovative countries ranked by our approach we focused on countries’ performances in relation to the fifth determinant listed above, the development of R&D capability by the public and private sector. For this purpose we analyzed the proportion of publications addressing at least one of the 17 NTDs listed by the World Health Organization [27].

**Fig 2:**
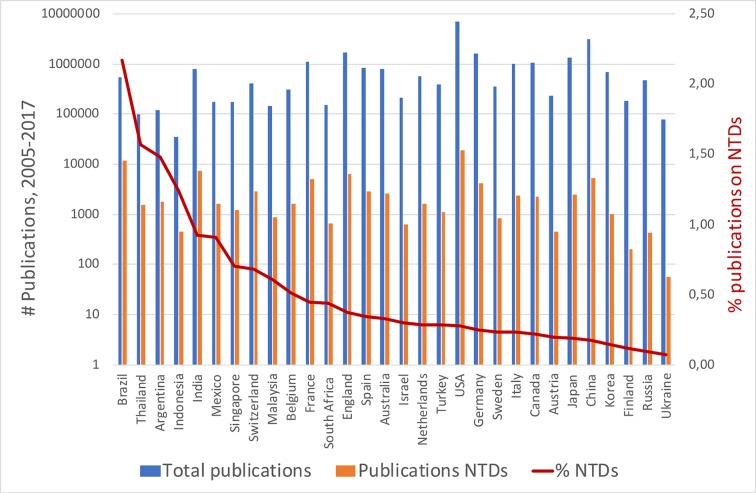
Distribution of countries according to % publications addressing NTDs. The red line spans from 0.07% (Ukraine) up to 2.17% (Brazil).

Fig 2 ranks the top 30 innovative countries according to the proportion of their scientific publications addressing at least one NTD in the abstract. On the average, 0.53% of their publications addresses at least one NTD. The following countries publish above this average (IDCs in italics): *Brazil (2.17%), Thailand (1.57%), Argentina (1.48%), Indonesia (1.24%), India (0.92%), Mexico (0.91%)*, Singapore (0.70%), Switzerland (0.68%) and *Malaysia (0.61%)*.

### IDCs and epidemics

Two serious epidemics hit the developing world in 2014-2016, leading the World Health Organization to issue alerts of Public Health Emergency of International Concern (PHEIC) on two occasions: Ebola in West Africa in 2014 [28] and Zika in South America in 2016 [29].

**Fig 3:**
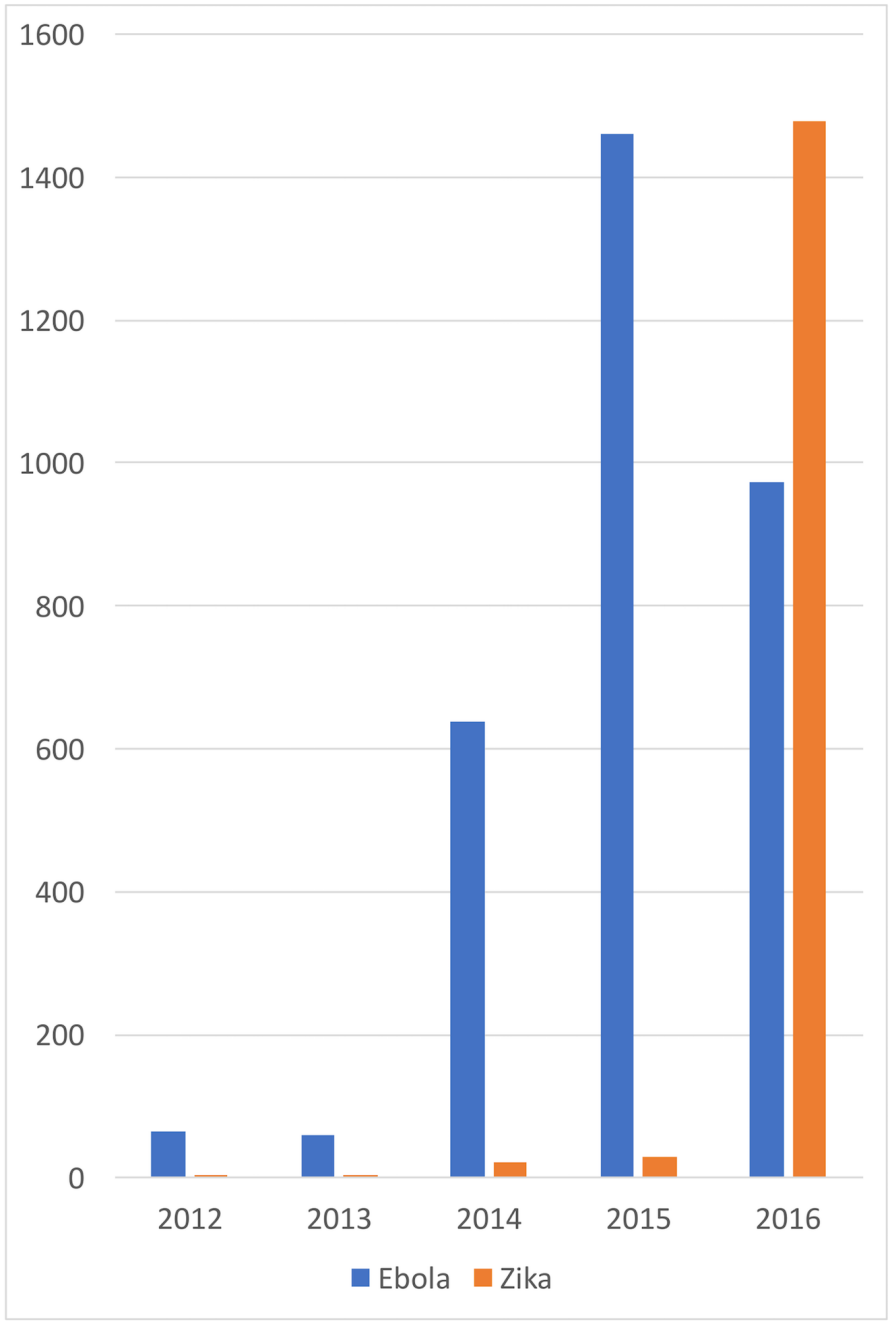
Evolution of publications on Ebola and Zika, 2012-2016. Publications on Ebola were already non negligible before the epidemics and peaked in 2015 while the Zika virus was not really in the global radar screen of researchers or institutions before the epidemics spread in Brazil in 2015.

Fig 3 shows the evolution of publications of scientific articles having “Ebola” or “Zika” in the title from January 2012 to December 2016.

We investigated the role of IDCs in these major public health emergencies analyzing coauthorship networks of scientific publications as previously described [12–14]. The analysis of co-authorship networks through social network analysis (SNA) has been applied previously to understand scientific collaboration in NTDs in Brazil [12–14], Canada [30] and Germany [31].

Co-authorship network analysis allows better understanding of the markedly cooperative context in which scientific knowledge is generated by identifying, among other information, key leading members (countries, organizations or individuals) that could act as “bridges” in the scientific community. Network analysis includes quantitative metrics addressing properties of network members, estimating the importance of a node relative to all other nodes in a given network, taking into account the different ways in which it interacts and communicates with the rest of the network. Centrality measures are the most commonly used to identify the nodes that have strategic significance in the network [16]. Particularly useful are the betweenness centrality values of individual nodes, indicating whether they are connecting parts of a network that would only be poorly connected or not connected at all. Nodes with high betweenness centrality are called bridges, brokers or boundary spanners for their ability to facilitate access to novel information, or resources, facilitate transfer of knowledge, and co-ordinate effort across the network [32]. They are considered key players in that their loss from a network would greatly affect its function and viability, and can be regarded as innovation hubs within networks [33, 34].

**Fig 4:**
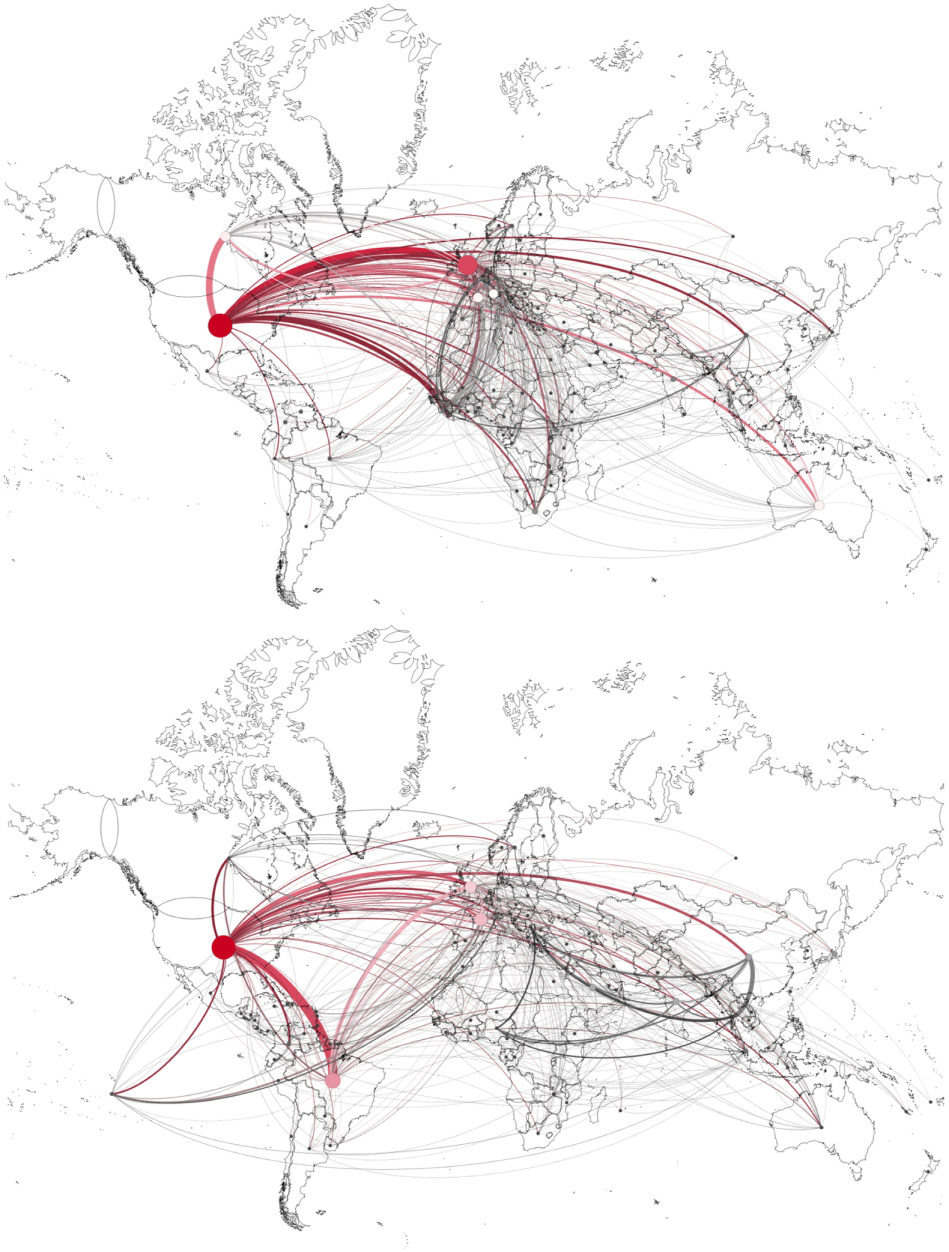
Coauthorship country networks addressing epidemics. Each node represents one country and two countries were considered connected if their authors shared the authorship of a paper. The thickness of links indicates the frequency of collaboration between two nodes. Bigger sizes and warmer colors indicate high betweenness centrality. Upper part: countries publishing on Ebola, 2015. Lower part: countries publishing on Zika, 2016.

Fig 4 displays the coauthorship networks addressing the epidemics of Ebola (2015) and Zika (2016) while Table 2 lists the countries and organizations that are network cutpoints, the number of papers they published on the epidemics and their betweenness centrality measures. In the Ebola 2015 network only industrialized countries and their organizations played a relevant role. In contrast, the Zika 2016 network showed two Brazilian institutions, the Oswaldo Cruz Foundation (Fiocruz) and the University of São Paulo (USP) among the top 5. Brazil as a country—an IDC—had the 2nd strongest betweenness centrality, behind the US but ahead of the three OECD countries, France, UK and Italy.

**Table 2:** Coauthorship networks, Ebola 2015 and Zika 2016. Top 5 relevant countries and institutions, number of publications and betweenness centralities.

Ebola 2015			Zika 2016		
Country	Number of Articles	Betweenness Centrality	Country	Number of Articles	Betweenness Centrality
USA	650	0.2294	USA	508	0.2890
UK	173	0.1835	*Brazil*	220	0.1704
Canada	84	0.0772	France	81	0.1363
Australia	46	0.0736	UK	104	0.1217
France	89	0.0727	Italy	55	0.0501
Institution	Number of Articles	Betweenness Centrality	Institution	Number of Articles	Betweenness Centrality
Univ. London	73	0.1458	Univ. London	46	0.1704
WHO	57	0.1427	*Fiocruz*	72	0.1420
CDC USA	87	0.1117	Institut Pasteur	28	0.1332
Univ. California	43	0.0930	CDC USA	68	0.1195
NIH USA	80	0.0769	*Univ. São Paulo*	43	0.1048

Countries and institutions were ranked according to decreasing betweenness centrality measurements. IDCs and IDC institutions are displayed in italics. CDC USA = Centers for Disease Control & Prevention, United States; Fiocruz = Oswaldo Cruz Foundation, Brazil; NIH USA = National Institutes of Health, United States; WHO = World Health Organization, Switzerland.

## Discussion

### IDCs, a decade later

The introduction of the IDC concept a decade ago suggested that some developing countries were mobilizing their scientific and technological workforce to address the main health problems affecting their populations. In some of these countries this investment in health innovation was accompanied by a strong growth of their GNI per capita from 2003 to 2015—7x for China, 4x for Brazil and 3x for India. It is interesting to note that the leading IDCs on the right side of Table 1 (present study), include the BRICS, a group which has been improving its scientific excellence [35], but now Turkey is also among them, a country that together with Russia, did not show up among the top-25 in the original study [2].

### Measuring the innovative performance and capacity of countries

Different approaches have been used to evaluate and measure the innovative performance, capacity and potential of countries. The Global Innovation Index (GII) “was launched in 2007 with the simple goal of determining how to find metrics and approaches that better capture the richness of innovation in society and go beyond such traditional measures of innovation as the number of research articles and the level of research and development (R&D) expenditures” [36]. The Bloomberg Innovation Index (BII) “rates countries on seven factors that when used together, are a representation of innovation levels” [37].

Table 3 lists the top 25 most innovative countries according to these two approaches and to the methodology adopted in this study. It is worth noting that in the BII and GII ranks is the presence of two, or just one country, respectively, that do not belong to the high income economy category—China and Malaysia (21st and 23rd positions) on the BII and China (22nd position) on the GII. This is in stark contrast not only to this study’s rankings, which include eight IDCs among the top 25 countries, with China topping the very first position, but also to recent economic analyses indicating the prominent economic and technological role of China today [38, 39].

**Table 3:** Top 25 innovative countries according to different indexes. Countries’ nomenclature according to the original studies. Non-high income economies (IDCs) are shown in italics.

#	Bloomberg Innovation Index (2017) [40]	Global Innovation Index (2017) [41]	This study Index PCT/GNI per capita
1	South Korea	Switzerland	*China*
2	Sweden	Sweden	Japan
3	Germany	Netherlands	USA
4	Switzerland	USA	*India*
5	Finland	UK	Republic of Korea
6	Singapore	Denmark	Germany
7	Japan	Singapore	France
8	Denmark	Finland	United Kingdom
9	U.S.	Germany	Netherlands
10	Israel	Ireland	Italy
11	France	Republic of Korea	*Russian Federation*
12	Austria	Luxembourg	*Turkey*
13	Belgium	Iceland	Sweden
14	Norway	Japan	Canada
15	Netherlands	France	*Brazil*
16	Ireland	Hong Kong (China)	Spain
17	UK	Israel	*South Africa*
18	Australia	Canada	Switzerland
19	New Zealand	Norway	Ukraine
20	Canada	Austria	Israel
21	*China*	New Zealand	Finland
22	Poland	*China*	*Mexico*
23	*Malaysia*	Australia	Austria
24	Italy	Czech Republic	*Malaysia*
25	Iceland	Estonia	Australia

Why such large discrepancies? One explanation resides on the conceptual framework behind each methodology: While BII and GII use multiple activities, parameters, indicators and weights to estimate innovative capacity and performance, we focused on the number of total international patent applications (PCT), normalized for each country’s economic strength and population, as a proxy to assess its innovation capacity.

**Fig 5:**
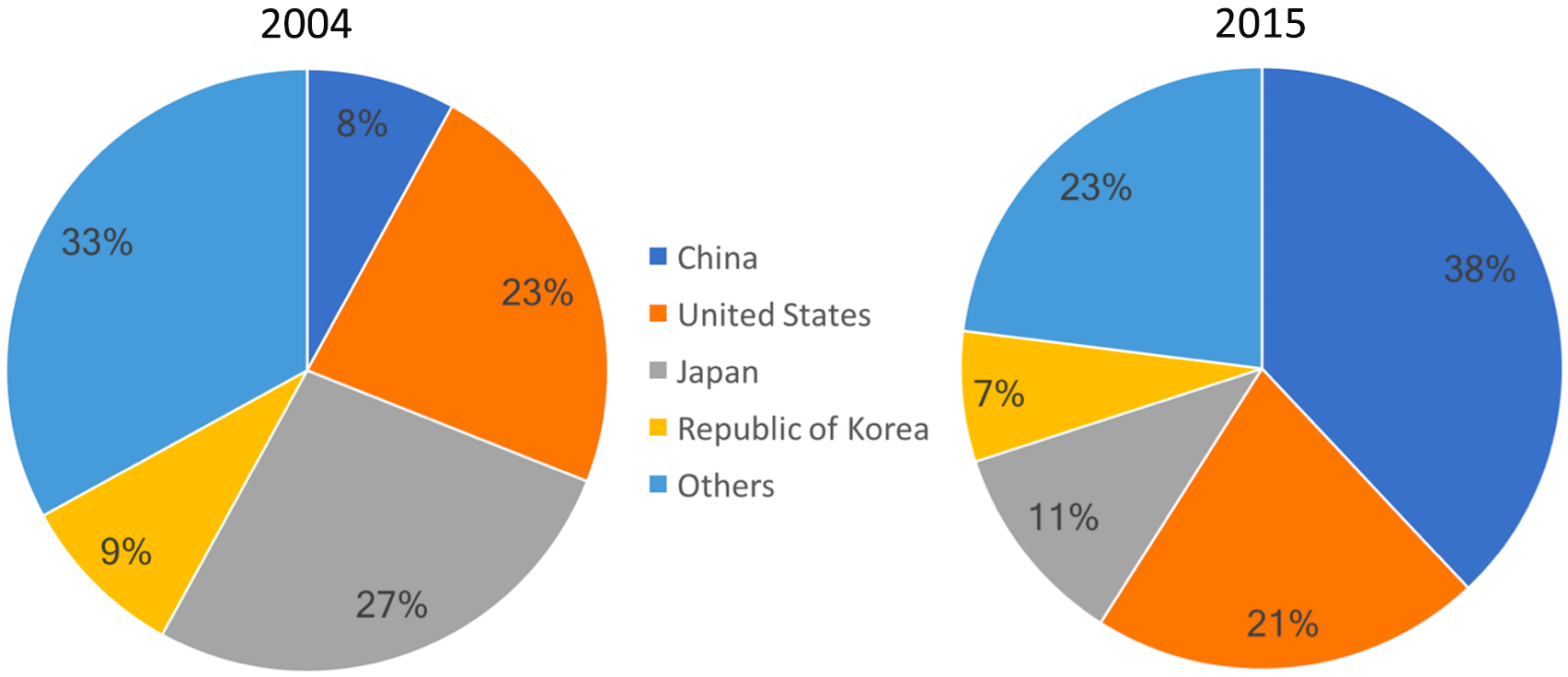
Distribution of total patent applications in the world, 2004/2015. The top 4 country patent offices are indicated. Source: WIPO Statistics (World 2004: 1.574.200 patent applications; World 2015: 2.888.800 patent applications). Available in: https://www3.wipo.int/ipstats/keysearch.htm?keyId=221. Access in: August 18, 2017.

The prominent innovative role of China today is not yet fully recognized mainly due to the small penetration of the Chinese language in international technological and scientific databases. This language barrier, however, is becoming less important due to the technological evolution of machine translation systems. A partnership involving the European Patent Office and Google, for example, led to the creation of a new technology called Neural Machine Translation (NMT) [42]. NMT has allowed a large quantity of patent documents previously restricted to a country’s own patent office and language to become freely available in international databases. In 2015 China became the first country office to receive over a million patent applications in a single year, receiving almost as many applications as United States, Japan and Republic of Korea combined [43] (Fig 5). In this way technologies developed in China and patented in Chinese became freely available for consultation in other languages, disclosing to the rest of the world the recent technological progress of that country.

Each ranking approach carries its own pros and cons, advantages and disadvantages and will therefore generate a different type of classification. Our approach relies on patent statistics, an indicator for science and technology directly relevant to innovation measurements, easily available in an entrepreneurial environment and whose importance is recognized by the OECD [44]. Although using patents as a proxy to innovation is an ongoing debate [45, 46], our results strongly suggest that innovation should not be regarded as a privilege of high income, industrialized economies and that IDCs, particularly China and India, have become now serious players among the big actors.

### IDCs and NTDs

The origin of the “neglected diseases” concept may be traced to the Rockefeller Foundation’s Program “The Great Neglected Diseases of Mankind” founded in 1977 by Kenneth Warren, the Foundation’s Director of Medical Sciences. In his view diseases such as schistosomiasis, malaria and others were neglected by funding agencies such as the US National Institutes of Health which invested mostly in other diseases such as cancer [47, 48]. During the late 90s’ “neglected diseases” and “most neglected diseases” were regarded as neglected by the pharmaceutical companies as they had no interest in developing drugs or medicines for patients suffering from them [49, 50].

Several international initiatives were created and implemented in order to compensate for this “neglect” from funding agencies or pharmaceutical companies. One of the first was the UNICEF/UNDP/World Bank/WHO Special Programme for Research and Training in Tropical diseases (TDR), launched in 1975 and hosted at the World Health Organization in Geneva. In 2000 the United Nations Millennium Declaration and the establishment of the Millennium Development Goals was accompanied by the creation of several Partnerships for Product Development (PDPs) such as Medicines for Malaria Venture (MMV), the Global Alliance for Tuberculosis Drug Development (TB Alliance), the Foundation for Innovative New Diagnostics (FIND) and the Drugs for Neglected Diseases initiative (DND*i*).

An important outcome of this global mobilization was the recognition that NTDs have poverty-promoting features and other socioeconomic consequences; in other words they not only occur in the setting of poverty, they actually promote poverty [51]. This shift from a passive definition (neglected by someone else) to an active one (poverty promotion) brought the NTDs to the center of the developing countries’ social and economic development agendas, forcing the health and science and technology systems of IDCs to play a more endogenous, autonomous and active role in NTD control and prevention [3], instead of just waiting for medical solutions developed abroad, which was the main paradigm of the last century [52].

Fig 2 shows that IDCs invest above the average on NTDs in a critical component, or determinant, of health innovation: research and development [2, 22, 23]: Seven of the nine countries prioritizing NTDs R&D, are IDCs: Brazil, Thailand, Argentina, Indonesia, India, Mexico and Malaysia. The two industrialized countries that this study demonstrated that are also part of this top group, Singapore and Switzerland, have built and run two institutions fully dedicated to R&D on NTDs: the Novartis Institute for Tropical Diseases (NITD) in Singapore and the Swiss Tropical and Public Health Institute (Swiss TPH) in Basel.

### IDCs and the prevention and control of epidemics

Another important conceptual paradigm shift that continues to evolve is the focus “from neglected diseases to neglected populations” [53]. We used this point of view to analyze the role of IDCs and their institutions in two recent sanitary crises that impacted neglected populations in Africa and South America between 2014-2016: the Ebola epidemics in West Africa and the spread of Zika virus in South America.

Fig 4 and Table 2 illustrate that most of the work by researchers, epidemiologists and public health decision makers, as reflected by their publications in international peer reviewed journals that had the words “Ebola” or “Zika” in the title, had quite different profiles in terms of the geographic location of the authors and the relevance of the affected countries in coauthorship networks. During an epidemics, the position occupied by countries, institutions and authors in a network that is generating knowledge about a disease is an important parameter for influencing response, decision-making, preparedness and empowerment. In co-authorship networks the betweenness centrality of a country or institution can be an excellent indicator of this reality. A node with a higher betweenness centrality would have more control over the network due to the volume of information that will depend on that entity for the pass through of knowledge [54]. As a point of control in the communication network, betweenness centrality measures the degree to which a node can function and does not necessarily correlate with volume of publications. In the Ebola network, for example, Australia had higher betweenness centrality than France, but it was behind it in the number of publications (Table 2). In the Zika network, the UK, despite publishing more papers than France on this specific subject (104 and 81 papers, respectively), ranked lower in betweenness centrality.

The majority of the work conducted in West Africa to detect, diagnose and control the Ebola epidemics was carried out by teams brought from abroad in response to a dramatic appeal from the Director General of the World Health Organization when she declared the Ebola epidemics a Public Health Emergency of International Concern (PHEIC). On that occasion it was emphasized that West African countries’ health systems needed international help to manage infection [55].

The Zika epidemics in the Americas that started in the northeast of Brazil, on the other hand, was detected and characterized by physicians and researchers working at local health services, hospitals or universities. Brazilian scientists were responsible for seminal work on outbreak characterization [56–59], clinical case definition [60], sexual transmission [61]. Furthermore, their research was critical to document the anomalous high incidence of microcephaly and other newborn malformations that were associated them with Zika virus infecting pregnant women [62–66], and precipitated studies on antiviral treatment [67, 68] and vector biology [69, 70].

When a network of international scientists issued an alarming statement to propose that the “Rio de Janeiro’s 2016 Olympic Games must not proceed…because Brazil’s Zika problem is inconveniently not ending” [71], it was a report from Brazilian scientists [72] that quickly brought evidence that the epidemics had already receded. Based on facts, the WHO issued a formal press release stating that “there is no public health justification for postponing or cancelling the games” [73]. This decision proved correct: the Rio Olympics, which represented an investment of above 10 billion US dollars [74], proceeded as expected and no Zika cases being reported during the event [75].

### Limitations

It is known that patents are not strictly direct indicators of the innovation process. Nevertheless, as patents are a legal property right over an invention that provides to its owner an exclusive right for a limited period, patents are often issued along the route leading to innovation. In accordance with the OSLO manual (2005) patent statistics never ceased to be an especially relevant science and technology indicator to measure of technologies of product and process innovation.

We also recognize the limitation of using coauthorship data as a proxy of scientific collaboration, acknowledging that not all cooperative efforts result in publications, and not all co-authored papers necessarily infer collaboration and knowledge exchange. Even so, it is assumed that, in most cases, coauthorship indicates an active collaboration that goes beyond mere data sharing.

### Conclusions

Since its introduction in 2005, the IDC concept has positively contributed to the analysis of the roles that different countries play in innovation. Our present analysis, based on the recent Ebola and Zika epidemics, demonstrated the importance of the preexisting healthcare infrastructure and research networks in an IDC to mount an effective response against an emerging health threat. The overall response to the Ebola epidemic, which only affected non-IDC countries, was primarily driven by outside experts who were severely constrained by local customs and societal norms. This observation has significant global policy implications for future responses: the global community clearly needs to prioritize long-term support to strengthen local leadership in countries that encompass geographic hotspots of disease emergence [76]. Expanding the science, technology and innovation base in these countries and regions will improve the response to emerging disease outbreaks and is well aligned with reaching the United Nations Sustainable Development Goals (https://sustainabledevelopment.un.org/). We anticipate that the application of the IDC concept to areas beyond healthcare will uncover the participation of these countries in social and economic development, which traditional analytic tools have underestimated or not considered.

## Supporting information

S1 Table Original data, 2003. Reproduction of the information published by Morel et al 2005 [2].
(PDF)

S2 Table Top 25 innovative countries. Comparison of the 2005 original country ranking with those of the present study.
(PDF)
