# Supplementary material for: Revisiting the concept of Innovative Developing Countries (IDCs) for its relevance to health innovation and neglected tropical diseases and for the prevention and control of epidemics
Source: PLoS Negl Trop Dis. 2018 Jul 12;12(7):e0006469. doi: 10.1371/journal.pntd.0006469 (PMC6042684; doi:10.1371/journal.pntd.0006469)
Supplement: S1 Table — Reproduction of the information published by Morel et al 2005 [2]. (PDF) [file pntd.0006469.s001.pdf]

Supporting information 1

S1 Table. Original data, 2003 Reproduction of the information published by Morel et al 2005 [2]

Table 1. US patents, GDP/capita, and US patents/GDP/capita (2003)

#	Country	US patents	GDP/capita (US dollars)	US patents/ GDP/capita
1	USA	99,386	36,006	2.760
2	Japan	37,779	31,407	1.203
3	*India*	444	487	0.912
4	*China*	724	989	0.732
5	Germany	13,110	24,041	0.545
6	Korea, Rep.	4,246	10,006	0.424
7	France	4,682	24,061	0.195
8	Canada	4,410	22,777	0.194
9	UK	4,803	26,445	0.182
10	Italy	2,206	20,528	0.107
11	Israel	1,392	15,592	0.088
12	*Brazil*	209	2,593	0.081
13	Sweden	1,771	26,929	0.066
14	*South Africa*	142	2,299	0.062
15	Australia	1,174	20,822	0.056
16	Switzerland	1,845	36,687	0.050
17	Belgium	998	23,749	0.042
18	Finland	1,009	25,295	0.040
19	Austria	753	19,749	0.038
20	*Thailand*	64	2,060	0.031
21	*Argentina*	76	2,797	0.027
22	Singapore	564	20.886	0.027
23	*Malaysia*	95	3,905	0.024
24	*Mexico*	129	6,320	0.020
25	*Indonesia*	16	817	0.020
