# Supplementary material for: Revisiting the concept of Innovative Developing Countries (IDCs) for its relevance to health innovation and neglected tropical diseases and for the prevention and control of epidemics
Source: PLoS Negl Trop Dis. 2018 Jul 12;12(7):e0006469. doi: 10.1371/journal.pntd.0006469 (PMC6042684; doi:10.1371/journal.pntd.0006469)
Supplement: S2 Table — Comparison of the 2005 original country ranking with those of the present study. (PDF) [file pntd.0006469.s002.pdf]

Supporting information 2

S2 Table. Top 25 innovative countries. Comparison of the 2005 original country ranking with those of the present study.

Table 1. Top 25 innovative countries.

#	Morel et al, 2005 [1] USPTO 2003 per GDP per capita	This study USPTO 2015 per GDP per capita	This study PCT 2015 per GDP per capita	This study PCT 2015 per GNI per capita
1	USA	*India*	*China*	*China*
2	Japan	USA	Japan	Japan
3	*India*	*China*	USA	USA
4	*China*	Japan	*India*	*India*
5	Germany	Republic of Korea	Republic of Korea	Republic of Korea
6	Republic of Korea	Germany	Germany	Germany
7	France	Canada	France	France
8	Canada	UK	UK	UK
9	UK	France	*Russian Federation*	Netherlands
10	Italy	Israel	Netherlands	Italy
11	Israel	Italy	Italy	*Russian Federation*
12	*Brazil*	Netherlands	Sweden	*Turkey*
13	Sweden	*Russian Federation*	*Turkey*	Sweden
14	*South Africa*	*Brazil*	Canada	Canada
15	Australia	Ukraine	*Brazil*	*Brazil*
16	Switzerland	*Mexico*	Ukraine	Spain
17	Belgium	Sweden	Spain	*South Africa*
18	Finland	Spain	*South Africa*	Switzerland
19	Austria	Belgium	Switzerland	Ukraine
20	*Thailand*	Switzerland	Israel	Israel
21	*Argentina*	Australia	Finland	Finland
22	Singapore	*Philippines*	Austria	*Mexico*
23	*Malaysia*	Austria	*Mexico*	Austria
24	*Mexico*	*Malaysia*	*Malaysia*	*Malaysia*
25	*Indonesia*	Finland	Belgium	Australia

The first column shows the original 2005 country ranking, obtained by dividing the number of USPTO patent applications of each country by the country's GDP per capita. The second column is an update of the first one using USPTO 2015 patent information. The rankings in the third and fourth columns were obtained using the approach of the present study - PCT applications per GDP, or per GNI, per capita. Countries which are not considered high income economies by the World Bank (GNI per capita higher than US$ 12,476) and fit the IDC category are displayed in italics. S2 Table.
